# A PER3 Polymorphism Interacts with Sleep Duration to Influence Transient Mood States in Women

**DOI:** 10.5334/jcr.135

**Published:** 2016-03-10

**Authors:** Tatiana D. Viena, Christina M. Gobin, Ana I. Fins, Travis J.A. Craddock, Aurélien Tartar, Jaime L. Tartar

**Affiliations:** Department of Psychology and Neuroscience, Nova Southeastern University, Ft. Lauderdale, FL USA; Department of Clinical Psychology, Nova Southeastern University, Ft. Lauderdale, FL USA; Clinical Systems Biology Group, Institute for Neuro-Immune Medicine, Nova Southeastern University, Ft. Lauderdale, FL USA; Department of Computer Science, Nova Southeastern University, Ft. Lauderdale, FL USA; Department of Clinical Immunology, Nova Southeastern University, Ft. Lauderdale, FL USA; Department of Biological Sciences, Nova Southeastern University, Ft. Lauderdale, FL USA

**Keywords:** Anxiety, Depression, Mood, PER3, Sleep

## Abstract

**Background:** Expression of the clock family of genes in the suprachiasmatic nuclei (SCN) regulates the molecular control of circadian timing. Increasing evidence also implicates clock gene activity in the development of mood disorders. In particular, variation in the PER3 clock gene has been shown to influence diurnal preference and sleep homeostasis. However, there is not currently a clear association between PER3 polymorphisms and mood. This is possibly because the PER3 gene has been shown to influence homeostatic sleep drive, rather than circadian timing, and the PER3 gene may be behaviorally relevant only under chronic sleep loss conditions.

**Methods:** To test the association between PER3 allele status and impaired mood, a total of 205 healthy women were genotyped for PER3 allele status and responded to previously-validated psychological questionnaires surveying self-reported sleep habits (MEQ, PSQI) and mood. Our mood measures included two measures of short-term, transient mood (state anxiety and mood disturbance) and two measures of longer term, ongoing mood (trait anxiety and depressive symptomology).

**Results:** The PER3 genotype distribution was 88 (42.9%) for PER3(4/4), 98 (47.8%) for PER3(4/5), and 19 (9.3%) for PER3(5/5). Our sleep duration x genotype interaction analyses showed that, relative to longer allele carriers, PER3(4/4) genotypes were at greater risk for transient psychological effects (mood and state anxiety) when they reported reduced sleep durations.

**Conclusion:** Sleep duration plays a critical role in understanding the extent to which PER3 allele status relates to mood states.

## Introduction

Circadian rhythmicity is tightly regulated by clock genes such as PERIOD (PER) 1/2/3, Circadian Locomotor Output Cycles Kaput (CLOCK), cryptochrome (cry) 1/2, casein kinase 1ε (CK1ε) and brain muscle ARNT-like 1 (BMAL). Beyond regulating circadian rhythmicity, clock gene variations have also been implicated in mood disorders and changes in emotion processing. For example, polymorphisms in BMAL and PER3 were found to be significantly associated with bipolar disorder [1], CRY1 polymorphisms has been associated with major depression [2], and a mutation in the CLOCK gene results in decreased anxiety and mania [3]. The clock genes are expressed in the suprachiasmatic nuclei (SCN), and the general role of the SCN in mediating mood is reflected in the finding that bilateral lesion of the SCN has antidepressant effects in rats [4,5]. It is not completely surprising that variations in SCN genes are associated with mood changes since circadian misalignment and sleep/wake disturbances are common in patients suffering from mood disorders [6,7,8]. For example, 80–90% of patients with Major Depressive Disorder suffer from sleep disturbances (primarily insomnia) [6,7,9,10]. Indeed, therapy aimed at circadian rhythm stabilization in bipolar patients, where sleep/wake cycles are better controlled, helps to improve mood and overall daytime functioning [11].

While clock genes have been implicated in mood disorders, the extent to which they can influence changes in mood in a non-clinical population is less certain. The PER3 clock gene is a promising gene candidate for influencing daytime mood. The PER3 protein product forms a heterodimer with the CRY protein that translocates into the nucleus to inhibit CLOCK-BMAL-mediated transcription. The completion of this feedback loop takes ~24 hours to complete. Given that the PER3 gene has an 18 amino acid-long 4 and 5 repeat homolog, size differences in the PER3 protein could ostensibly change the timing of the circadian feedback loop. We specifically targeted this functional 4 and 5 variable number tandem-repeat (VNTR) polymorphism in the coding region of the PER3 gene (rs57875989) since a possible interaction between sleep duration and the PER3 genotype on mood has recently been suggested. In particular, the PER3 VNTR has been shown to influence the antidepressant effects of sleep deprivation [12] and impacts depressive symptoms in a geriatric population [13].

PER3-mediated changes in mood are possibly related to the influence of this gene on sleep duration. For example, relative to the PER3(4/4) genotype, the PER3(5/5) genotype is associated with shorter sleep durations during work days [14]. The PER3(5/5) allele carriers are also more likely to experience greater sensitivity to the adverse consequences of experimental sleep deprivation, while the PER(4/4) carriers demonstrate higher sensitivity during the recovery period following long duration sleep deprivation [15,16,17,18,19]. In addition, when the PER3 VNTR is introduced into mice, homeostatic sleep drive is affected with the PER3(4/4) genotypes being more vulnerable to the cognitive consequences of sleep loss [20]. This finding is reflected in human studies where the PER3 VNTR genotypes were found to be related to individual differences in neurobehavioral performance after sleep deprivation [21,22] and chronic sleep restriction [19,23].

Taken together, these findings suggest that sleep duration should be considered when examining the impact of PER3 genotype on mood. To that end, the present study aimed to find the interaction between PER3 allele status, chronic sleep restriction, and mood. We specifically tested women since, relative to men, the effects of sleep debt accumulate more quickly in women and sleep complaints in women are particularly associated with impaired psychological functioning [24,25]. We hypothesized that the PER3(4/4) allele status would be particularly associated with impaired mood among individuals displaying short sleep durations (≤6 hours of sleep).

## Methods

### Participants

Two hundred and twenty one women participants were recruited through flyers posted in public buildings and through the Nova Southeastern University (NSU) introductory psychology course participant pool. Participants who responded “yes” to demographic questions related to taking medication that altered sleep patterns, current or prior mental health illness, sleep difficulties, or diagnosis of a sleep related disorder in the past six months were excluded from further analysis. As a result of this screening, sixteen participants who self-reported taking medication that alters sleep were excluded. Self-reported race/ethnicities out of the remaining two hundred and five participants (mean age = 21.11, SD = 4.27) were as follows: 63 Non-Hispanic white, 75 Hispanic White, 30 Black/African-American, 18 Asian, and 19 Multiracial. All participants were compensated with either a $10 store gift card or research participation credit. Exclusion criteria included being younger than 18 years of age or over 50 years of age, having a positive history of mental illness, taking medication for sleep, or a diagnosed sleep disorder. The protocol approval was procured through the NSU Institutional Review Board (IRB).

### Procedure

All participants signed a written consent form, a demographic/general questionnaire and completed a series of psychological instruments to measure sleep and mood. Our sleep duration measure was taken from a fill-in-the-blank item in the Pittsburgh Sleep Quality Index (PSQI) [26] and was based on behavior over the previous month. This is in line with the established period of 30 days for diagnoses of chronic sleep disorders [27]. Our mood measures included two measures of short-term, transient mood (state anxiety and mood disturbance) and two measures of longer term, ongoing mood (trait anxiety and depressive symptomology).

The State-Trait Anxiety Inventory (STAI) was used to measure state and trait anxiety. The STAI has been used extensively in research and clinical practice and it has been validated as an accurate measure of anxiety in adults [28]. The Profile of Mood States (POMS) was applied to measure ongoing mood [29]. The POMS questionnaire is a valid and reliable instrument used to measure psychological distress (mood changes) in healthy, ill and psychiatric patients with a coefficient alpha of .84 or above [30]. The Center for Epidemiologic Studies Depression Scale (CES-D) was employed to measure depressive symptomatology [31]. Unlike other depression scales that focus on clinical depression, the CES-D [31] is frequently utilized with non-clinical samples. Detailed descriptions of the psychometric properties of the psychological instruments can be found elsewhere [42]. After completing the questionnaires, all participants provided 2 buccal swabs for DNA testing from the left and right cheeks. Swabs were stored at room temperature until DNA extraction was performed.

Once all samples were collected, genomic DNA extraction was performed using the QIAamp DNA Investigator kit, following the manufacturer instructions (QIAGEN, Valencia, CA). After isolation, a first round of amplification was conducted by polymerase chain reaction (PCR) using the primers PER3 F (5’-CAAAATTTTATGACACTACCAGAATGGCTGAC-3’) and PER3 R (5’-AACCTTGTACTTCCACATCAGTGCCTGG-3’), as previously described [32]. PCR conditions corresponded to the following pattern repeated for 30 cycles: 95 °C for 30 seconds, 50 °C for 30 seconds, and 72 °C for 1 minute. Aliquots corresponding to the products from the first round of amplification were used as templates for a second round of amplification (30 cycles, with the same conditions). The nested primers PER F2 (5’-GAAGATTAAAGTGTCTTTTCATGTGCCCTTAC-3’) and PER R2 (5’-AATGTCTGGCATTGGAGTTTGAAACATTAG-3’) used in the second reactions have been described by Ebisawa and colleagues [32]. Products of the nested PCR reactions were visualized on agarose gels (1% w./v.), and each participant’s genotype type was identified.

## Statistical Analyses

In order to test the hypothesis that PER3 genotype and sleep duration would have a combined impact on mood, univariate 2X2 ANOVA’s were used to compare the effect of PER3 genotype (4 and 5 repeat allele carriers) and sleep duration (≤6 hours of sleep, >6 hours of sleep) on the four independent measures of mood: the CES-D, state anxiety (STAI), trait anxiety (STAI), and the POMS total mood disturbance score. We combined the PER3(4/5) and PER3(5/5) genotypes into one group due to the ubiquitous shortage of the homozygous 5-repeat allele and to isolate the effects of the 4 repeat allele. This is consistent with previous studies that have investigated this PER3 polymorphism [33,34,35,36,37,38,39]. Our cutoff score of 6 hours or less for short sleep duration was chosen since the neurobehavioral consequences of sleep restriction begin to be observed at 6 hours of sleep (or time in bed) [40,41,42,43]. The distributions of four and five repeat allele frequencies were determined by the Hardy–Weinberg exact test, and the association of allele were analyzed using the chi-square test.

## Results

Subjects were identified by PER3 genotype using genotyping results (Table 1). PER3 genotype distribution was 88 (42.9%) for PER(4/4), 98 (47.8%) for PER3(4/5), and 19 (9.3%) for PER3(5/5). There were no significant differences in PER3 genotype across ethnicities (*F* (1,204) = 2.227, *p* = 0.137). The frequencies of genotypes deviates from the expected Hardy-Weinberg values (*χ*^2^ = *1.26; P = 0.26*) because of a ubiquitous shortage of the homozygous 5-repeat allele [14]. The data for all measures collected from all participants is included in Table 1.

**Table 1:** Characteristics of PER3(4/4), PER3(4/5), PER3(5/5) and PER3(–/5) Descriptive Variables.

Characteristic	Per 3 4/4		Per 3 4/5		Per 3 5/5		Per 3 –/5 Combined	
				
	Means	SD	Means	SD	Means	SD	Means	SD

**N**	88		98		19		117	
**Age**	21.10	3.85	21.14	4.59	21.00	4.67	21.12	4.58
**Ave Hours Sleep**	6.72	1.42	6.67	1.38	6.47	1.26	6.64	1.35
**Anxiety-State**	41.78	13.60	40.71	13.34	38.05	10.80	40.28	12.96
**Anxiety-Trait**	40.05	11.65	40.95	10.90	40.00	8.32	40.79	10.50
**PSQI Total score**	6.18	2.74	6.44	3.03	6.16	3.08	6.39	3.03
**POMS Depression**	7.16	9.57	6.84	8.92	5.89	7.84	6.68	8.73
**POMS Anger**	5.19	6.00	3.93	5.47	5.26	6.35	4.15	5.61
**POMS Tension**	8.98	7.42	8.80	7.39	7.95	6.45	8.66	7.22
**POMS Vigor**	11.48	7.35	11.48	6.32	9.79	6.53	11.21	6.36
**POMS Confusion**	7.02	4.71	6.69	4.58	5.79	4.93	6.55	4.63
**POMS Fatigue**	9.10	6.72	8.26	6.49	8.84	6.15	8.35	6.42
**POMS Mood Score**	25.98	30.82	22.95	28.59	23.95	29.68	23.11	28.65
**Depression CES-D**	14.61	9.71	14.18	8.71	10.94	7.20	13.68	8.54

*Note.* *PER3(–/5) Combined collapses PER3(4/5) and PER3(5/5).

**Figure 1:**
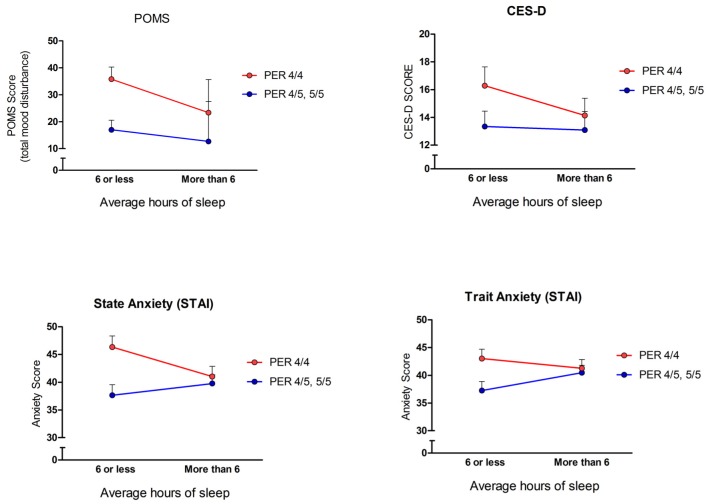
Relationship between sleep duration and mood impairment among PER3 groups. A main effect of sleep duration and total mood disturbance (POMS) was revealed (*F* (1,204) = 5.41, *p* = 0.02). A main effect of state anxiety (STAI-State) and sleep duration was also found (*F* (1,204) = 7.32, *p* = 0.007). There was no significant main effect of PER3 genotype alone on either the POMS or STAI measures (*p*’s > 0.05). Additionally, a significant interaction between PER3 genotype and sleep duration on the POMS scale (*F* (1,204) = 4.96, *p* = 0.03), and PER3 genotype and sleep duration on the state anxiety score (*F* (1,204) = 4.07, *p* = 0.04) was discovered. Neither depressive symptomatology (CES-D) nor trait anxiety (STAI-Trait) scores showed significant main effects of genotype or sleep duration or significant interaction effects (all *p*’s > 0.05). Values reflect means ± SEM.

Our hypothesis that there would be an interaction between short sleep duration and mood impairments in the PER3 short allele carrier group was partially supported. As shown in Figure 1, only the measures of transient mood states (STAI-state, POMS) showed significant genotype by mood assessment interactions. For the POMS measure, there was a significant main effect for self-reported average hours of sleep, *F (1,204) = 5.41, p = 0.02*. The group that slept 6 hours or less per night had greater total mood disturbance (*M = 29.58, SE = 3.06*) than the group who reported sleeping more than 6 hours per night (*M = 19.97, SE = 2.78*). There was not a significant main effect for PER3 genotype alone (*p > 0.05*). There was a significant interaction between the PER3 genotype and sleep duration on the POMS total mood disturbance score *F (1,204) = 4.96, p = 0.03*. On the STAI measure (state and trait anxiety), the state anxiety measure, reflecting general anxiety during the experimental testing situation, showed a significant main effect for sleep duration *F (1,204) = 7.32, p = 0.007*. In agreement with the POMS measure, those who report sleeping less than 6 hours per night had greater state anxiety (*M = 43.68, SE = 1.37*) than those who report sleeping more than 6 hours per night (*M = 38.69, SE = 1.24*). There was not a significant main effect for PER3 genotype on state anxiety scores (*p > 0.05*), but there was a significant interaction between PER3 genotype and sleep duration on state anxiety scores *F (1,204) = 4.07, p = 0.04*. Neither depressive symptomatology (CES-D) nor trait anxiety scores showed significant main effects of genotype or sleep duration or significant interaction effects (all *p’s > 0.05).*

## Discussion

The current study revealed that, in a sample of healthy women with no self-reported sleep dysfunction, relative to the longer allele carriers, the PER3(4/4) allele carriers were at greater risk for transient psychological effects when they reported reduced sleep (≤6 hrs/night). In particular, we showed that a reduced sleep duration, combined with the PER3(4/4) genotype, was associated with greater mood disturbances and increased state anxiety. This was not the case for depressive symptomatology or trait anxiety- there were no interactions between PER3 allele status and sleep duration on these measures.

Short sleep durations alone have been shown to predict mood disturbances [42], and in agreement, we found that short sleep durations relate to greater mood disturbance. A central goal of the current study was to use self-report sleep measures in an effort to capture those individuals who experience volitional ongoing reduced sleep duration. The majority of studies done on PER3 and sleep loss have focused on one or a few nights of total sleep deprivation [12,18,23,44] or sleep restriction under tight experimental conditions [19,45,46]. However, most people rarely experience total sleep deprivation or mandatory sleep restriction, but rather, experience volitional CSR, and tend to purposefully restrict their sleep time on a regular basis to meet the demands of daily life. To our knowledge, only one previous study has examined the relationship between volitional sleep behavior and PER3 allele type [21]. Our results suggest that the influence of PER3 allele status on mood is at least partially mediated by volitional sleep restriction.

It is possible that the PER3 VNTR effects on mood are mediated through the ongoing reduced sleep durations coupled with dysregulated internal circadian functions, such as body core temperature, melatonin, and cortisol levels [47]. A polymorphism in the PER3 gene has been associated with earlier wake time [48], whereas the PER3(4/4) genotype is associated with an evening preference and delayed sleep phase syndrome (DSPS) [49,50]. Individuals with DSPS experience a higher incidence of negative psychological problems such as nervousness, lack of emotional expression, and mood disorders. A relationship between PER3 and sleep may come about through altered homeostatic processes. Hasan and colleagues [20] showed that the PER3 VNTR influences sleep homeostatic processes (rather than circadian rhythm or diurnal preference) when a primate-specific gene was expressed in mice. Furthermore, molecular markers of cognitive vulnerability to sleep loss were also altered in the humanized PER3 VNTR in mice. Work in humans suggests that the PER3(5/5) carriers have increased sleep pressure, experience enhanced sleep initiation, and are vulnerable to the cognitive consequences of sleep deprivation [18].

We found that relative to the PER3 5 repeat allele carriers, the PER3(4/4) genotypes were more sensitive to transient psychological effects of short sleep. However, previous work has shown that, relative to the short allele carries, the PER3(5/5) genotypes demonstrate poorer performance of cognitive tasks after sleep deprivation [15,16,17]. An important distinction between our study and previous reports, however, is that we examined the interaction between the PER3 allele status and ongoing short sleep duration (not acute total sleep deprivation). In addition, we tested the effects of sleep duration and PER3 allele status on mood as opposed to cognitive performance measures. Finally, our study was restricted to a population of women, who are more at risk for mood disturbances relative to men [51].

One important consideration in the present study is that we restricted our study to healthy women, and as a consequence, our results may not be generalizable to other populations. Another possible limitation may have been the use of subjective measures to determine sleep and mood measurements. Furthermore, the psychological inventories utilized have been previously validated and shown to be highly reliable.

In sum, although there is a clearly demonstrated comorbidity between sleep behavior and mood disorders, the intricacies and mechanisms of this association remain poorly understood. Findings from this study help to shed light on the complex relationship between sleep and mood by showing how short sleep durations and PER3 allele status combine to predict psychological health.

## Competing Interests

The authors declare that they have no competing interests.

## References

[1] Nievergelt CM, Kripke DF, Barrett TB, Burg E, Remick RA, Sadovnick AD (2006). Suggestive evidence for association of the circadian genes PERIOD3 and ARNTL with bipolar disorder. American Journal of Medical Genetics Part B: Neuropsychiatric Genetics.

[2] Soria V, Martínez-Amorós È, Escaramís G, Valero J, Pérez-Egea R, García C (2010). Differential association of circadian genes with mood disorders: CRY1 and NPAS2 are associated with unipolar major depression and CLOCK and VIP with bipolar disorder. Neuropsychopharmacology.

[3] Roybal K, Theobold D, Graham A, DiNieri JA, Russo SJ, Krishnan V (2007). Mania-like behavior induced by disruption of CLOCK. Proceedings of the National Academy of Sciences.

[4] Arushanian E, Popov A (1994). [The effect of damage to the hypothalamic suprachiasmatic nuclei in rats on the dynamic short-period fluctuations of their normal and abnormal behaviors]. Fiziologicheskii zhurnal imeni IM Sechenova/Rossiiskaia akademiia nauk.

[5] ğTatarolu Ö, Aksoy A, Yılmaz A, Canbeyli R (2004). Effect of lesioning the suprachiasmatic nuclei on behavioral despair in rats. Brain Res.

[6] Armitage R (2007). Sleep and circadian rhythms in mood disorders. Acta Psychiatrica Scandinavica.

[7] Ford DE, Cooper-Patrick L (2001). Sleep disturbances and mood disorders: an epidemiologic perspective. Depression and anxiety.

[8] Wirz-Justice A (2006). Biological rhythm disturbances in mood disorders. International Clinical Psychopharmacology.

[9] Thase ME (2006). Depression and sleep: pathophysiology and treatment. Dialogues Clin Neurosci.

[10] Motivala SJ, Levin MJ, Oxman MN, Irwin MR (2006). Impairments in health functioning and sleep quality in older adults with a history of depression. J Am Geriatr Soc.

[11] Frank E, Swartz HA, Kupfer DJ (2000). Interpersonal and social rhythm therapy: managing the chaos of bipolar disorder. Biological psychiatry.

[12] Dallaspezia S, Locatelli C, Lorenzi C, Pirovano A, Colombo C, Benedetti F (2016). Sleep homeostatic pressure and PER3 VNTR gene polymorphism influence antidepressant response to sleep deprivation in bipolar depression. Journal of affective disorders.

[13] Maglione JE, Nievergelt CM, Parimi N, Evans DS, Ancoli-Israel S, Stone KL (2015). Associations of PER3 and RORA Circadian Gene Polymorphisms and Depressive Symptoms in Older Adults. The American Journal of Geriatric Psychiatry.

[14] Lázár AS, Slak A, Lo JC-Y, Santhi N, von Schantz M, Archer SN (2012). Sleep, diurnal preference, health, and psychological well-being: a prospective single-allelic-variation study. Chronobiol Int.

[15] Lo JC, Groeger JA, Santhi N, Arbon EL, Lazar AS, Hasan S (2012). Effects of partial and acute total sleep deprivation on performance across cognitive domains, individuals and circadian phase.

[16] Vandewalle G, Archer SN, Wuillaume C, Balteau E, Degueldre C, Luxen A (2009). Functional magnetic resonance imaging-assessed brain responses during an executive task depend on interaction of sleep homeostasis, circadian phase, and PER3 genotype. The Journal of Neuroscience.

[17] Groeger JA, Viola AU, Lo JC, von Schantz M, Archer SN, Dijk D-J (2008). Early morning executive functioning during sleep deprivation is compromised by a PERIOD3 polymorphism. Sleep.

[18] Maire M, Reichert C, Gabel V, Viola A, Strobel W, Krebs J (2014). Sleep ability mediates individual differences in the vulnerability to sleep loss: Evidence from a PER3 polymorphism. Cortex.

[19] Rupp TL, Wesensten NJ, Newman R, Balkin TJ (2013). PER3 and ADORA2A polymorphisms impact neurobehavioral performance during sleep restriction. J Sleep Res.

[20] Hasan S, Van der Veen DR, Winsky-Sommerer R, Hogben A, Laing EE, Koentgen F (2014). A human sleep homeostasis phenotype in mice expressing a primate-specific PER3 variable-number tandem-repeat coding-region polymorphism. The FASEB Journal.

[21] Viola AU, Archer SN, James LM, Groeger JA, Lo JC, Skene DJ (2007). PER3 polymorphism predicts sleep structure and waking performance. Curr Biol.

[22] Groeger JA, Viola AU, Lo J, von Schantz M, Archer SN, Dijk D-J (2008). Early morning executive functioning during sleep deprivation is compromised by a PERIOD3 polymorphism. Sleep.

[23] Lo JC, Groeger JA, Santhi N, Arbon EL, Lazar AS, Hasan S (2012). Effects of partial and acute total sleep deprivation on performance across cognitive domains, individuals and circadian phase. PloS one.

[24] Armitage R, Smith C, Thompson S, Hoffmann R (2001). Sex differences in slow-wave activity in response to sleep deprivation. Sleep Res Online.

[25] Voderholzer U, Al-Shajlawi A, Weske G, Feige B, Riemann D (2003). Are there gender differences in objective and subjective sleep measures? A study of insomniacs and healthy controls. Depress Anxiety.

[26] Buysse DJ, Reynolds CF, Monk TH, Berman SR, Kupfer DJ (1989). The Pittsburgh Sleep Quality Index: a new instrument for psychiatric practice and research. Psychiatry Res.

[27] American Psychiatric Association (2000). Electronic DSM-IV-TR plus.

[28] Okun A, Stein RE, Bauman LJ, Silver EJ (1996). Content validity of the Psychiatric Symptom Index, CES-depression Scale, and State-Trait Anxiety Inventory from the perspective of DSM-IV. Psychol Rep.

[29] McNair DM, Lorr M, Droppleman LF (1971). Manual for the Profile of Mood States.

[30] McNair D, Lorr M, Droppleman L (2013). Profile of Mood States.

[31] Radloff LS (1977). The CES-D scale a self-report depression scale for research in the general population. Applied psychological measurement.

[32] Ebisawa T, Uchiyama M, Kajimura N, Mishima K, Kamei Y, Katoh M (2001). Association of structural polymorphisms in the human period3 gene with delayed sleep phase syndrome. EMBO reports.

[33] Alexander M, Burch JB, Steck SE, Chen CF, Hurley TG, Cavicchia P (2015). Case-control study of the PERIOD3 clock gene length polymorphism and colorectal adenoma formation. Oncol Rep.

[34] Cerliani MB, Gili JA, Pavicic WH, Klein G, Saba S, Richard SM (2015). Association between PER3 length polymorphism and onco-hematological diseases and its influences on patients’ functionality. Advances in Modern Oncology Research.

[35] Dai H, Zhang L, Cao M, Song F, Zheng H, Zhu X (2011). The role of polymorphisms in circadian pathway genes in breast tumorigenesis. Breast cancer research and treatment.

[36] Guess J, Burch JB, Ogoussan K, Armstead CA, Zhang H, Wagner S (2009). Circadian disruption, Per3, and human cytokine secretion. Integrative cancer therapies.

[37] Yamaguchi M, Kotani K, Tsuzaki K, Takagi A, Motokubota N, Komai N (2015). Circadian Rhythm Genes CLOCK and PER3 Polymorphisms and Morning Gastric Motility in Humans. PloS one.

[38] Wirth MD, Burch JB, Hébert JR, Kowtal P, Mehrotra-Kapoor A, Steck SE (2014). Case–Control Study of Breast Cancer in India: Role of PERIOD 3 Clock Gene Length Polymorphism and Chronotype. Cancer Invest.

[39] Drake CL, Belcher R, Howard R, Roth T, Levin AM, Gumenyuk V (2015). Length polymorphism in the Period 3 gene is associated with sleepiness and maladaptive circadian phase in night-shift workers. J Sleep Res.

[40] Drake CL, Roehrs TA, Burduvali E, Bonahoom A, Rosekind M, Roth T (2001). Effects of rapid versus slow accumulation of eight hours of sleep loss. Psychophysiology.

[41] Van Dongen HP, Maislin G, Mullington JM, Dinges DF (2003). The cumulative cost of additional wakefulness: dose-response effects on neurobehavioral functions and sleep physiology from chronic sleep restriction and total sleep deprivation. SLEEP-NEW YORK THEN WESTCHESTER-.

[42] Dinges DF, Pack F, Williams K, Gillen KA, Powell JW, Ott GE (1997). Cumulative sleepiness, mood disturbance and psychomotor vigilance performance decrements during aweek of sleep restricted to 4–5 hours per night. Sleep: Journal of Sleep Research & Sleep Medicine.

[43] Vgontzas AN, Zoumakis E, Bixler E, Lin H-M, Follett H, Kales A (2004). Adverse effects of modest sleep restriction on sleepiness, performance, and inflammatory cytokines. The journal of Clinical Endocrinology & Metabolism.

[44] Viola AU, James LM, Archer SN, Dijk D-J (2008). PER3 polymorphism and cardiac autonomic control: effects of sleep debt and circadian phase. American Journal of Physiology-Heart and Circulatory Physiology.

[45] Goel N, Banks S, Mignot E, Dinges DF (2009). PER3 polymorphism predicts cumulative sleep homeostatic but not neurobehavioral changes to chronic partial sleep deprivation. PloS one.

[46] Möller-Levet CS, Archer SN, Bucca G, Laing EE, Slak A, Kabiljo R (2013). Effects of insufficient sleep on circadian rhythmicity and expression amplitude of the human blood transcriptome. Proceedings of the National Academy of Sciences.

[47] Kline CE, Durstine JL, Davis JM, Moore TA, Devlin TM, Youngstedt SD (2010). Circadian rhythms of psychomotor vigilance, mood, and sleepiness in the ultra-short sleep/wake protocol. Chronobiol Int.

[48] Lee KA, Gay C, Byun E, Lerdal A, Pullinger CR, Aouizerat BE (2015). Circadian regulation gene polymorphisms are associated with sleep disruption and duration, and circadian phase and rhythm in adults with HIV. Chronobiol Int.

[49] Archer SN, Robilliard DL, Skene DJ, Smits M, Williams A, Arendt J (2003). A length polymorphism in the circadian clock gene Per3 is linked to delayed sleep phase syndrome and extreme diurnal preference. Sleep.

[50] Jones KH, Ellis J, Von Schantz M, Skene DJ, DIJK DJ, Archer SN (2007). Age-related change in the association between a polymorphism in the PER3 gene and preferred timing of sleep and waking activities. Journal of sleep research.

[51] Seney ML, Sibille E (2014). Sex differences in mood disorders: perspectives from humans and rodent models. Biology of sex differences.

